# Nociceptin Signaling Involves a Calcium-Based Depolarization in *Tetrahymena thermophila*

**DOI:** 10.1155/2013/573716

**Published:** 2013-04-29

**Authors:** Thomas Lampert, Cheryl Nugent, John Weston, Nathanael Braun, Heather Kuruvilla

**Affiliations:** ^1^Department of Biological Sciences, State University of New York at Buffalo, 109 Cooke Hall, Buffalo, NY 14260, USA; ^2^Department of Science and Mathematics, Cedarville University, 251 North Main Street, Cedarville, OH 45314, USA

## Abstract

*Tetrahymena thermophila* are free-living, ciliated eukaryotes. Their behavioral response to stimuli is well characterized and easily observable, since cells swim toward chemoattractants and avoid chemorepellents. Chemoattractant responses involve increased swim speed or a decreased change in swim direction, while chemorepellent signaling involves ciliary reversal, which causes the organism to jerk back and forth, swim in small circles, or spin in an attempt to get away from the repellent. Many food sources, such as proteins, are chemoattractants for these organisms, while a variety of compounds are repellents. Repellents in nature are thought to come from the secretions of predators or from ruptured organisms, which may serve as “danger” signals. Interestingly, several peptides involved in vertebrate pain signaling are chemorepellents in *Tetrahymena*, including substances P, ACTH, PACAP, VIP, and nociceptin. Here, we characterize the response of *Tetrahymena thermophila* to three different isoforms of nociceptin. We find that G-protein inhibitors and tyrosine kinase inhibitors do not affect nociceptin avoidance. However, the calcium chelator, EGTA, and the SERCA calcium ATPase inhibitor, thapsigargin, both inhibit nociceptin avoidance, implicating calcium in avoidance. This result is confirmed by electrophysiology studies which show that 50 *μ*M nociceptin-NH2 causes a sustained depolarization of approximately 40 mV, which is eliminated by the addition of extracellular EGTA.

## 1. Introduction

Nociceptin/orphanin FQ (hereafter referred to as nociceptin) is a peptide involved in vertebrate pain signaling. The endogenous receptor for this ligand is ORL-1/NCR [1, 2]. A number of signaling pathways have been implicated in vertebrate nociceptin signaling. A partial listing of molecules involved in this signaling cascade would include G_i/o_ proteins [1], neuronal nitric oxide synthase (nNOS) [3], and Erk-dependent signaling [4]. In addition, signaling through the nociceptin receptor induces a reduction in calcium influx via P/Q-type calcium channels in rat brain [5]. 

*Tetrahymena thermophila* are free-living, unicellular eukaryotes. While *T. thermophila* do not feel pain, they are capable of sensing chemoattractants and chemorepellents in their environment. This allows them to find food and possibly to escape predation [6]. A recent review by Csaba [7] details the response of *T. thermophila* to a number of chemoattractants and chemorepellents, including their response to many vertebrate hormones. Indeed, *T. thermophila *appear to synthesize and respond to a number of vertebrate hormones, including serotonin, melatonin, adrenocorticotropic hormone, and insulin [7].

A number of chemorepellents which have been characterized in *T. thermophila* are polycationic peptides, including lysozyme [8], the lysozyme fragment CB2 [9], PACAP [10], and nociceptive peptides including bradykinin and substance P [11]. Lysozyme signaling involves a calcium-based depolarization [12]. Lysozyme and PACAP appear to share a signaling pathway [9], which involves cAMP and phospholipase C [13], as well as NOS and cGMP [14]. A related peptide, VIP, also uses these signaling pathways and cross-adapts with lysozyme and PACAP, suggesting that *Tetrahymena* are signaling through a generalized polycation receptor [15]. 

Nociceptin is a polycationic peptide that is commercially available in three different isoforms. Nociceptin carries a charge of +4 at pH 7.0, while nociceptin-NH2 carries a charge of +5 at pH 7.0. Nociceptin-Arg_14_Lys_15_ carries a charge of +6 at pH 7.0. Our hypothesis was that all three of the nociceptin analogues would be chemorepellents in *T. thermophila *and that more highly charged nociceptin isoforms will have a lower EC_100_ in behavioral assays than isoforms which carry a lesser charge.

## 2. Materials and Methods

### 2.1. Cell Cultures

*Tetrahymena thermophila*, strain B2086, a generous gift from Hennessey [6] (SUNY Buffalo), was used for all of the experiments. Cells were grown at 25°C in the axenic medium of Dentler [16], without shaking or addition of antibiotics. Two-day old cell cultures were used for all behavioral assays described below.

### 2.2. Chemicals and Solutions

Behavioral assays were carried out in a buffer of pH 7.0 containing 10 mM Trizma base, 0.5 mM MOPS, and 50 *μ*M CaCl_2_. All repellents and inhibitors used were dissolved in this buffer.

All nociceptin isoforms, thapsigargin, J-113397, and EGTA, were purchased from Tocris Biosciences, Bristol, UK. 

### 2.3. Behavioral Assays

Behavioral assays were carried out as previously described [8, 10, 17]. Ten milliliters of *T. thermophila* culture was washed by centrifugation in a clinical centrifuge at high speed, and the pellet was reconstituted in 10 mL buffer. This wash step was repeated twice, and cells were reconstituted in 5 mL of buffer for use in behavioral assays. To perform the behavioral assays, 300 *μ*L of cell suspension was transferred to the first well of a microtiter plate. Cells were then transferred individually using a micropipette into the second well of the microtiter plate, which contained 300 *μ*L of buffer as a control. Cells were then transferred to a third well containing 300 *μ*L of nociceptin. Behavior of the cells was observed for the first 5 seconds after transfer to the third well, and the percentage of cells exhibiting avoidance behavior was noted. Varying concentrations of each peptide were used until we determined the minimum concentration at which 90% of the cells exhibited avoidance behavior (EC_100_). Each trial represents 10 cells. A minimum of 6 trials was performed for each data point.

Pharmacological inhibition assays were performed similar to the behavioral assays described previously. After being washed in buffer, cells were exposed to pharmacological agents known to block specific signaling pathways and incubated for 15 minutes to 2 hours. Cells were then transferred to a solution containing nociceptin at EC_100_ and then monitored for avoidance behavior. Each trial represents 10 cells. A minimum of 6 trials was performed for each data point.

Cross-adaptation assays were performed as previously described [10, 17]. Briefly, 300 *μ*L of cells were placed into the first well of a 3-well microtiter plate. Cells were then individually transferred to the second well of the 3-well microtiter plate, which contained a repellent. The cells were allowed to adapt to this repellent for 10–15 minutes or until cells showed baseline avoidance (an avoidance of no more than 20%). Cells were then individually transferred to the third well of the 3-well microtiter plate, which contained the repellent to be tested for cross-adaptation and monitored for avoidance behavior. Data which showed 20% or fewer cells exhibiting avoidance was considered “baseline avoidance.” Baseline avoidance is the number of cells in our assay which show avoidance behavior when being transferred from one well containing buffer to another well containing the same buffer and usually ranges from 5 to 20%. Cells exhibiting baseline avoidance in response to this assay were considered to be cross-adapted. Each trial represents 10 cells. A minimum of 6 trials was performed for each data point.

### 2.4. Electrophysiology

Standard one-electrode whole-cell membrane potential recordings were recorded as the previously reported procedures in *Tetrahymena thermophila *[9, 12]. The recording buffer contained were carried out in a buffer of pH 7.0 containing 10 mM Trizma base, 0.5 mM MOPS, and 1 mM CaCl_2_. Membrane potentials were displayed on a digital oscilloscope and retained on a chart recorder during continuous bath perfusion at a rate of approximately 20.0 mL/min. The recording bath had a volume of approximately 1 mL. Solutions were changed by switching valves connected either to buffer or to the experimental solution without changing the flow rate of the perfusion system.

## 3. Results

**Figure 1:**
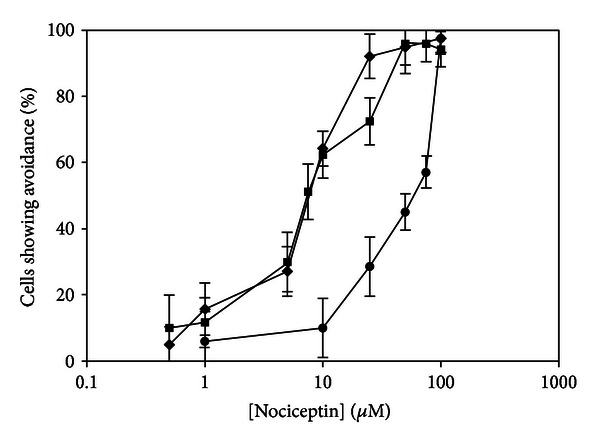
Nociceptin is a chemorepellent in *Tetrahymena thermophila*. Nociceptin (closed circles), nociceptin-NH2 (closed squares), and nociceptin-Arg_14_Lys_15_ (closed diamonds) all caused avoidance in *Tetrahymena thermophila*. The EC_100_ of each compound was correlated with its charge. Nociceptin, which has a net charge of +4, had an EC_100_ of 100 *μ*M. Nociceptin-NH2, which has a net charge of +5, had an EC_100_ of 50 *μ*M. Finally, nociceptin-Arg_14_Lys_15_, which had a net charge of +6, had an EC_100_ of 25 *μ*M. *N* ≥ 6. *N* represents the number of trials conducted. Each trial consisted of 10 cells, which were individually scored as positive or negative for avoidance.

All isoforms of nociceptin were chemorepellents in *T. thermophila* (Figure 1). Nociceptin, which has a charge of +4 at our assay pH of 7.0, had an EC_100_ of 100 *μ*M in our behavioral assay. Nociceptin-NH2, which has a charge of +5 under assay conditions, had an EC_100_ of 50 *μ*M, while nociceptin-Arg_14_-Lys_15_ which has a charge of +6 under assay conditions had an EC_100_ of 25 *μ*M. Avoidance was observed for 1–5 seconds but was seen for as long as 10–15 minutes (not shown). After cells acclimated to the nociceptin, they returned to forward swimming.

Cross-adaptation assays (Table 1) show that all three isoforms of nociceptin cross-adapt with one another. However, nociceptin-adapted cells did not cross-adapt to PACAP-38, and PACAP-adapted cells did not cross-adapt to nociceptin. Since all three nociceptin isoforms cross-adapted to one another, implying a common signaling pathway, we used 50 *μ*M nociceptin-NH2 in all subsequent pharmacological and behavioral assays. 

**Figure 2:**
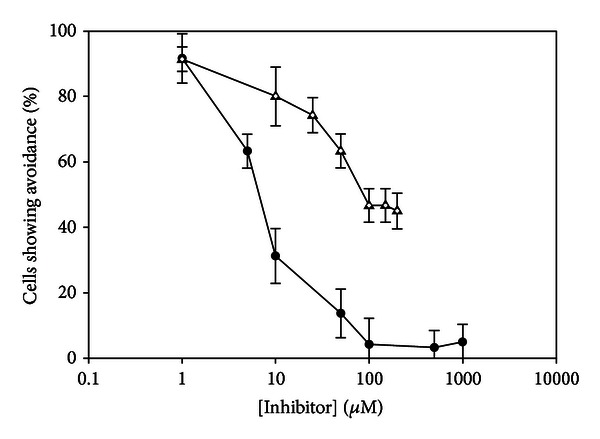
Calcium chelators inhibit the behavioral response to 50 *μ*M nociceptin-NH2 in *Tetrahymena thermophila*. EGTA (closed circles) reduces avoidance to 20% (near baseline) at a concentration of 50 *μ*M. The IC_50_ of EGTA is approximately 7.5 *μ*M. Thapsigargin (open triangles) reduced avoidance by 50% at a concentration of 100 *μ*M; however, increasing the concentration to 300 *μ*M did not cause a significant decrease in avoidance beyond that seen with 100 *μ*M thapsigargin. *N* ≥ 6. *N* represents the number of trials conducted. Each trial consisted of 10 cells, which were individually scored as positive or negative for avoidance.

Studies with pharmacological agents known to block G-protein signaling, tyrosine kinase signaling, and broad spectrum kinase activity had no effect on avoidance behavior in *T. thermophila *(Table 2). However, studies with the calcium chelator, EGTA, and the SERCA ATPase inhibitor, thapsigargin, both affected nociceptin avoidance (Figure 2). A concentration of 50 *μ*M EGTA was sufficient to reduce avoidance to a baseline avoidance of 20%. Thapsigargin, however, never reduced avoidance to baseline under the conditions of our assay. The highest concentration of thapsigargin we were able to achieve in our assay was 300 *μ*M. Thapsigargin reduced avoidance by 50% at a concentration of 100 *μ*M; however, increasing the concentration to 300 *μ*M did not decrease avoidance beyond that seen with 100 *μ*M thapsigargin.

**Figure 3:**
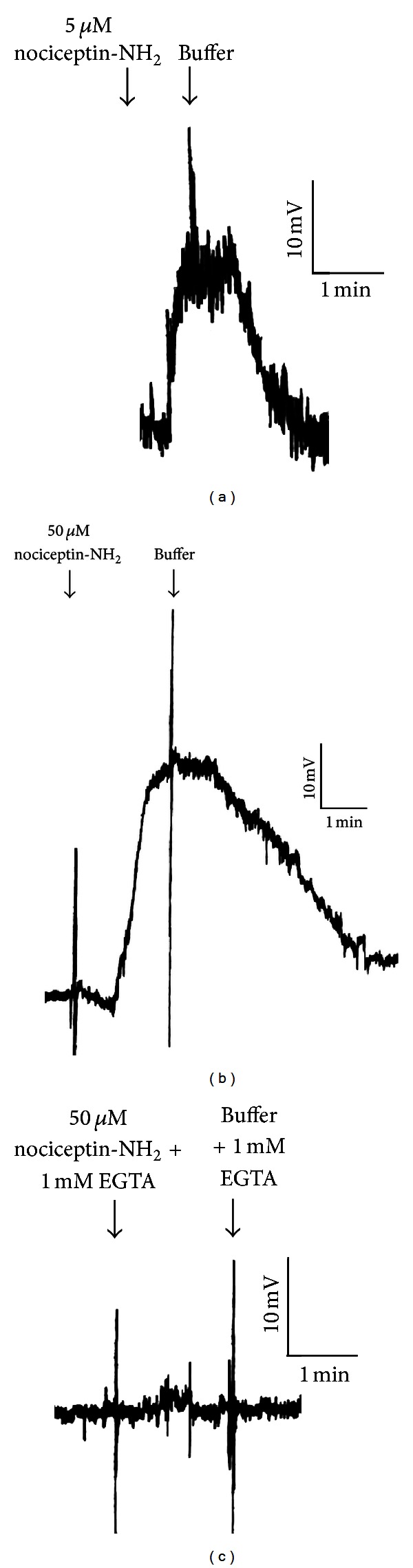
Nociceptin-NH2 is a depolarizing signal in *Tetrahymena thermophila*. (a) 5 *μ*M nociceptin-NH2 causes a depolarization of approximately 20 mV, though this concentration does not often provoke a behavioral response in *Tetrahymena. *(b) 50 *μ*M nociceptin-NH2 causes a depolarization of approximately 40 mV. This concentration is the EC_100_ for behavioral avoidance in *Tetrahymena.* (c) The depolarization produced by 50 *μ*M nociceptin-NH2 is eliminated by the addition of 1 mM EGTA to the external medium, implying that calcium is involved in the depolarization.

Whole-cell electrophysiology studies indicate that nociceptin-NH2 is a depolarizing signal in *T. thermophila* (Figure 3). A nociceptin-NH2 concentration of just 5 *μ*M was sufficient to elicit a depolarization of approximately 20 mV, though this concentration does not cause behavioral avoidance above baseline levels in *Tetrahymena *(Figures 3(a) and 1).Fifty *μ*M nociceptin-NH2, which is the EC_100_ for behavioral avoidance in *Tetrahymena*, elicited a depolarization of approximately 40 mV (Figure 3(b)). The depolarization produced by 50 *μ*M nociceptin-NH2 was eliminated by the addition of 1 mM EGTA to the external medium (Figure 3(c)).

**Figure 4:**
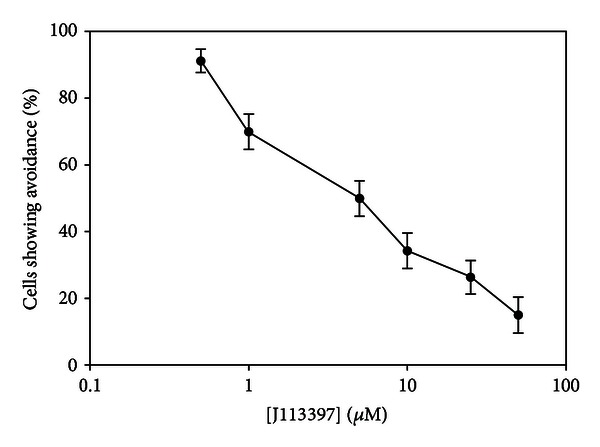
J-113397, a competitive inhibitor of the human nociceptin receptor, inhibits the behavioral response to 50 *μ*M nociceptin-NH2 in *Tetrahymena thermophila *when applied extracellularly. The IC_50_ of this compound is approximately 5 *μ*M. *N* ≥ 6. *N* represents the number of trials conducted. Each trial consisted of 10 cells, which were individually scored as positive or negative for avoidance.

J-113397, a competitive inhibitor of the human nociceptin receptor, inhibited the behavioral response to 50 *μ*M nociceptin-NH2 in *Tetrahymena thermophila *when applied extracellularly (Figure 4). Baseline avoidance to nociceptin was achieved by the addition of 50 *μ*M of J-113397.

## 4. Discussion

Our results confirmed our hypothesis that all three nociceptin isoforms tested would serve as chemorepellents in *T. thermophila *(Figure 1). In addition, the EC_100_ of each compound was correlated with the charge, with the most highly charged isoform having the lowest EC_100_, although all of the EC_100_ values were in a similar range. The correlation of lower EC_100_ values with a higher charge is consistent with what we have seen using other charged peptides in *T. thermophila. * For example, when we have used various peptides derived from ACTH, the more highly charged peptides caused avoidance at lower concentrations than did the less highly charged peptides [11]. In addition, our previous studies with PACAP and VIP [15] show that PACAP is effective at causing avoidance at a 1000-fold lower concentration than VIP, though presumably acting through the same receptor and/or signaling pathway. The isoform of PACAP that we used in the 2003 study, PACAP-38-NH2, has a net charge of +11 at pH 7.0, while VIP has a net charge of just +4 at the same pH. While factors other than charge are certainly involved in the interaction between these peptides and their putative receptor, it is highly probable that charge is playing a role in these interactions, possibly by increasing the affinity of ligand for its receptor. In the case of nociceptin, the charge differences were relatively small as were the differences in EC_100_.

Cells acclimated to nociception within 10–15 minutes of first being exposed to it (not shown). All isoforms of nociceptin were cross-adapted to one another, indicating that all forms of nociceptin were using the same receptor and/or signaling pathway. This is similar to what has previously been shown for lysozyme [8] and PACAP/VIP [15]. Since PACAP, lysozyme, and VIP appear to share a common receptor [10, 15], we cross-adapted cells to nociceptin and PACAP to determine whether nociceptin was using the same receptor/signaling pathway as the three previously studied polycationic ligands. As Table 1 shows, PACAP-adapted cells did not cross-adapt to nociceptin and nociceptin-adapted cells did not cross-adapt to PACAP. This indicates that nociceptin signals through a pathway that does not involve the previously described polycation receptor.

The previously studied PACAP response appears to be mediated through a G-protein-coupled receptor which uses adenylyl cyclase, phospholipase C, and nitric oxide synthase [10, 13–15]. In order to further ascertain whether nociceptin was using a separate signaling pathway, we used pharmacological inhibitors to block G-protein-linked receptors and associated pathways. None of these inhibitors blocked avoidance to nociceptin (Table 2), giving further evidence that the previously described polycation receptor is not being used in nociceptin signaling. This also differs from the vertebrate nociceptin receptor, which signals through G_i/o_ proteins [1].

Since a tyrosine kinase has been implicated in GTP signaling in *T. thermophila* [18] as well as insulin signaling [19], we also tested a battery of protein kinase and tyrosine kinase inhibitors to determine whether nociceptin signaling would be inhibited. None of these inhibitors affected nociceptin signaling (Table 2). Interestingly, genomic studies of *Tetrahymena* [20] show no evidence of the presence of a tyrosine kinase in this organism. 

Since a calcium-based depolarization is elicited by the addition of lysozyme [12] as well as the lysozyme fragment, CB_2_ [8], to *T. thermophila, *we wished to determine whether calcium was involved in nociceptin signaling in this organism. Studies with the external calcium chelator, EGTA (Figure 2) indicated that extracellular calcium was necessary for behavioral avoidance to nociceptin, since concentrations of EGTA above 50 *μ*M reduced avoidance down to baseline. Baseline avoidance in this organism is determined by counting the number of cells that show avoidance behavior when transferred from one well of buffer to another well of the same buffer [10]. The SERCA ATPase inhibitor, thapsigargin, was used to determine whether internal calcium stores were required in order for avoidance to occur. As seen in Figure 2, exposure of cells to 100 *μ*M thapsigargin reduced avoidance by approximately 50%. However, the avoidance response was not completely inhibited, indicating that while intracellular calcium may play a role in avoidance, lack of intracellular calcium stores depleted by thapsigargin may be partially compensated for by allowing extracellular calcium into the cytosol. Notably, the thapsigargin concentration used in this study was much higher than what we used in a previous study [18], in which only 1 nM thapsigargin was necessary in order to block the behavioral response to GTP. This is further evidence that extracellular calcium is primarily responsible for nociceptin avoidance. Calcium is not necessary for avoidance to all peptides, however, since avoidance of netrin-1, semaphorin 3C, and fragments of ACTH is unaffected by addition of either EGTA or thapsigargin [11].

Whole-cell electrophysiology studies indicate that nociceptin causes a depolarization in *T. thermophila* (Figure 3), even at concentrations that normally do not cause a behavioral response in this organism (Figures 3(a) and 1). When the EC_100_ of nociceptin-NH2 was used, the amplitude of the depolarization increased (Figure 3(b)). Finally, we were able to remove the depolarization by the addition of EGTA to the external medium (Figure 3(c)), implying that calcium is involved in the depolarization. This is similar to the previously described responses to lysozyme [12] and the lysozyme fragment, CB2 [9]. 

The involvement of calcium in nociceptin avoidance in *T. thermophila* is rather different from the human response to nociceptin, which involves closing calcium channels [5]. However, we did use J-113397, which is a competitive inhibitor of the human nociceptin receptor [21], in order to determine if it could also block *T. thermophila* avoidance to nociceptin. As shown in Figure 4, 50 *μ*M J-113397 was effective in reducing avoidance to baseline. This drug had no effect on avoidance to ACTH fragments (data not shown), suggesting that the response was specific to nociceptin. While we have not identified the receptor or signaling pathway that nociceptin is using in *T. thermophila*, these data suggest that there may be commonalities between the human nociceptin receptor and a possible nociceptin-binding protein in *T. thermophila.*

In summary, we have shown that nociceptin is a chemorepellent in *Tetrahymena* which elicits a depolarization. It does not act through the previously described polycation receptor nor does it signal through a G-protein-mediated receptor like the vertebrate nociceptin receptor. However, the J113397 studies imply that *Tetrahymena *may possess some type of receptor that shares binding characteristics with the human nociceptin receptor. Further studies may help elucidate the signaling mechanisms used in nociceptin avoidance in *T. thermophila*. If the receptor is identified, comparisons between the human nociceptin receptor and the unknown nociceptin-sensing mechanism in *T. thermophila* would be instructive. 

## 5. Conclusions

The vertebrate signaling peptide, nociceptin, is a chemorepellent in *Tetrahymena thermophila.* The effectiveness of signaling is impacted by the charge of the nociceptin isoform, with more highly charged forms of nociceptin requiring lower concentrations to signal effectively. Nociceptin does not signal through the previously described polycation receptor of *Tetrahymena thermophila* nor does it signal through a G-protein-linked receptor, as it does in humans. However, nociceptin avoidance in *Tetrahymena thermophila *is blocked by addition of J-113397, a competitive inhibitor of the vertebrate nociceptin receptor. This suggests that the vertebrate nociceptin receptor and its analog in *Tetrahymena* may share common binding characteristics. Finally, nociceptin signaling provokes a depolarization, which pharmacological studies suggest may be caused by an influx of calcium.

## Figures and Tables

**Table 1:** Nociceptin cross-adaptation studies. Cells were adapted to a given ligand by incubating them in that ligand for 10–15 minutes or until avoidance behavior ceased. Cells were then moved into another ligand and were scored positively or negatively for avoidance. Cross-adaptation with various analogues of nociceptin all show avoidance values that are at or below baseline (≤20%; [10]). However, cross-adaptation with the polycationic peptide, PACAP, does not cross-adapt with nociception, implying that nociception is using a pathway that is distinct from the previously described lysozyme/PACAP receptor [10, 15]. *N* represents the number of trials conducted. Each trial consisted of 10 cells, which were individually scored as positive or negative for avoidance.

	Nociceptin	Nociceptin-NH_2_	Nociceptin Arg_14_Lys_15_	PACAP 1-38
Nociceptin	9.2 ± 8.2	5 ± 8.3	0 ± 0	96.6 ± 5.8
	*N* = 13	*N* = 6	*N* = 6	*N* = 6
Nociceptin-NH_2_	5 ± 7.5	16.9 ± 12.2	14.5 ± 12.4	100 ± 0
	*N* = 9	*N* = 8	*N* = 12	*N* = 6
Nociceptin Arg_14_Lys_15_	3.3 ± 5.8	13.3 ± 12.1	16.6 ± 16.3	91.25 ± 9.9
	*N* = 6	*N* = 6	*N* = 6	*N* = 8
PACAP 1-38	97.5 ± 4.6	100 ± 0	100 ± 0	13.3 ± 5.8
	*N* = 8	*N* = 10	*N* = 10	*N* = 6

**Table 2:** Pharmacological inhibitors which act on G-protein mediated receptor pathways and tyrosine kinase pathways do not significantly impact nociception avoidance. *N* represents the number of trials conducted. Each trial consisted of 10 cells, which were individually scored as positive or negative for avoidance.

Pharmacological inhibitor	Pathway inhibited	Percentage of cells avoiding nociceptin	*N*
Control	None	94.28 ± 5.34	8
50 *μ*M RpcAMPs	Adenylyl cyclase	96.67 ± 5.16	6
1 mM GDP-*β*-S	G-proteins	100.0 ± 0.0	6
1 *μ*M U-73122	Phospholipase C	97.78 ± 4.40	9
1 *μ*M U-73345	Inactive analogue of U-73122	96.67 ± 5.16	6
10 *μ*M calphostin C	Protein kinase C	96.0 ± 5.0	10
1 mM 1400 W	NOS	96.0 ± 5.0	10
100 *μ*M tyrphostin 47	Receptor tyrosine kinases	92.5 ± 9.57	6
100 *μ*M AG126	Map kinase pathway	96.0 ± 6.99	10
150 *μ*M SU 6668	Receptor tyrosine kinases	91.66 ± 4.08	6
300 *μ*M apigenin	Protein kinases	91.2 ± 6.4	8
3 mM H-9	Protein kinases	96.6 ± 5.1	6

## Abbreviations

ACTH: Adrenocorticotropic hormone
EC_100_: Concentration of chemorepellent that causes avoidance in 100% of individuals
EGTA: Ethylene glycol tetraacetic acid
IC_50_: Concentration of inhibitor that blocks 50% of avoidance
PACAP: Pituitary adenylyl cyclase activating polypeptide
VIP: Vasoactive intestinal peptide.
